# Costing of actions to safeguard vulnerable Mexican households with young children from the consequences of COVID-19 social distancing measures

**DOI:** 10.1186/s12939-020-01187-3

**Published:** 2020-05-19

**Authors:** Mireya Vilar-Compte, Víctor Pérez, Graciela Teruel, Aranzazu Alonso, Rafael Pérez-Escamilla

**Affiliations:** 1 Research Institute for Equitable Development EQUIDE, Universidad Iberoamericana, Prolongación Paseo de la Reforma 880, Lomas de Santa Fe, ZIP 01219 Mexico City, Mexico; 2 Pacto por la Primera Infancia, Mexico City, Mexico; 3 Yale School of Public Health, New Haven, Connecticut USA

**Keywords:** COVID-19, Costing, Mexico, Childhood well-being

## Abstract

COVID-19 has imposed unprecedented challenges to society. As the pandemic evolves, the social distancing measures that have been globally enforced, while essential, are having undesirable socioeconomic side effects particularly among vulnerable populations. In Mexico, families who depend upon informal employment face increased threats to their wellbeing, and households who in addition have young children may face long-term consequences. The Mexican government has not yet taken actions, but a coalition of non-governmental organizations is advocating in partnership with academic institutions for social protection actions such as a cash transfer and basic services subsidies for families with young children, subsisting from the informal sector economy. To facilitate governmental action, we estimated the costs for implementation of these recommendations. The methodology used could be replicated in other countries facing similar challenges.

## Background

Stay home recommendations to enforce social distancing have been one of the key interventions to control the COVID-19 (SARS CoV-2, Coronavirus disease) pandemic. The Mexican Federal Government has gradually imposed a series of measures to contain the COVID-19 outbreak: schools’ closures (March 20), suspension of public events (March 23), suspension of all non-essential activities of the federal government (March 26), and suspension of all non-essential activities and voluntary self-isolation (March 30). While social distancing measures have been key to flattening the curve, its social and economic effects are unevenly distributed across societies – potentially affecting to a greater extent already vulnerable groups, particularly low-income households with children [1, 2].

Mexico is a highly unequal society with a large share of its population living in poverty and subsisting in the informal sector of the economy. This context poses major challenges to the success of the social distancing measures as many individuals cannot afford to remain at home. Because the unequal socio-economic impacts of the distancing measures might further exacerbate pre-existing inequalities it is key to implement appropriate social protection policies while the pandemic lasts. A large share of the Mexican population is employed in the informal economy with little social protection to cope with this crisis and with little if any employment security.

The negative effects of social distancing in the wellbeing of families with young children is especially worrisome due to the long-lasting effects it can have on health, cognitive and social development. Before the COVID-19 crisis hit, it was estimated that about 4.5 million households in Mexico had young children (0–5 years) with the head of household working in the informal economy. These households have a higher risk of facing major income reductions as well as household food insecurity [3] and household stress and risk of violence [4] as result of the social distancing measures. This in turn can reduce compliance with social distancing, and increases the risk of infection among household members and their communities.

While the Federal Government established some interventions for the protection of some vulnerable groups (i.e. older adults) as a result of COVID-19, inexplicably, it has not taken clear measures to protect vulnerable families with young children in spite of the fact that social interventions for these groups have been absent in this administration. In response to a lack of social protection actions in the face of COVID-19, the civil society coalition named El *Pacto por la Primera Infancia* – an advocacy and collective impact initiative, made up of more than 440 members, including civil society organizations, foundations and research institutions, united for the common purpose of making early childhood a national priority–led advocacy efforts to persuade the government to act. In partnership with other institutions, such as the Yale School of Public Health, it developed a list of action points for immediate implementation by the Federal Government:

1. Immediate implementation of a temporary program of non-conditional cash transfers aimed to protect families without social security, working in the informal sector and, with children under the age of 6.
2. A decree, to ensure that during the “social distancing” period, the delivery of services such as water, electricity, telephone, gas and internet shall not be suspended to families for nonpayment.
3. The offer of inclusive programming, in public-access television and online, to allow children and adolescents, to continue their studies of basic education, and to participate in a variety of tasks and activities at home aimed to reduce the stress of social distancing.
4. The implementation of an evidence-based social communication campaign, to promote self-care measures, mental health, nurturing and sensitive childcare and violence prevention.
5. To enable a telephone and online counseling program to protect the mental health of parents and caregivers, and to reduce the risk of domestic violence [5].

Partnering with Universidad Iberoamericana, two of these action points were costed: the cash-transfer (action point 1) and the subsidy for key services (i.e. water, electricity, telephone, gas, and Internet service) (action point 2). These actions were prioritized due to potentially being the costlier measures, as well as the need for immediate implementation to protect the well-being of these vulnerable families. By providing costing estimates, our aim was to help the government understand the budgetary needs of the proposed actions.

Our aim is to make available the costing methodology and estimates for the Mexican case to other countries, as they are based on commonly available survey data. This methodology may be used by other countries proposing similar interventions to protect vulnerable families with young children from the negative socio-economic impacts from social distancing measures required to tackle COVID-19.

## Main text

### Methods

To cost out the cash transfer and the subsidy (as a “nonpayment”) for key household services we used the National Income and Expenditure Survey 2018 (ENIGH, for its acronym in Spanish) [6]. As it is unclear how long would the stay-at-home requirements will last, we estimate their monthly cost. We use as proxy of informal employment lack of access to health services as a job benefit or working in firms with up to 5 employees. We also added households whose head was unemployed, since we assumed that under the current epidemic circumstances they would face considerable restrictions to look for a job.

For the cash-transfer, we estimated its cost based on one monthly minimum wage (approximately $154 US dollars), although this could be adapted to contextual estimates (e.g., poverty lines, which in the case of Mexico have a similar value to the minimum wage). In addition, we computed the estimates considering all “informal” households with children 0–5, as well as excluding those with older adults, as they were already receiving an extra COVID-related subsidy from the Federal Government (a 4-months upfront payment of the universal non-contributory pension program, totaling $218 US dollars). We further added a 3% administrative cost related to disbursing the transfer.

For the services subsidy (i.e. “nonpayment”), we estimated the cost based on the expenditures observed in ENIGH 2018 among households with the desired characteristics (i.e., those whose head works in the “informal” sector or is unemployed, have children aged 0–5, and have no older adults). As it is assumed that this subsidy would be a direct deduction from government-run companies or directly paid to the service providers, no extra administrative cost is added to this part.

## Results

According to the weighted estimates, 4,522,182 households would be eligible for the cash transfer and the services’ subsidy. The estimated cost of a monthly cash-transfer of one minimum wage would be about $715.6 million US dollars, including the administrative costs. In addition, the subsidy for water, electricity, telephone, gas and internet service would be approximately $91.5 million US dollars. In total, both interventions would have a monthly cost of $807.2 million US dollars (less than 0.06% of the GDP of Mexico).

### Suggestions for advocacy and implementation of the cash transfer

Mexican Legislation contemplates mechanisms to face emergencies like the current pandemic, through a Federal government declaration of a “state of emergency”, leading to the provision of extra financial resources to adopt policies like the ones costed. A relevant barrier that should be taken into consideration is the implementation process. A 3% administrative cost was considered based on the assumption that the government would use already existing structures to distribute the cash transfer such as schools, hospitals or community center. However, in light of the actions from the current government in dismantling pre-existing social programs like PROSPERA (the largest anti-poverty cash transfer) and the Seguro Popular (the universal public health insurance), there might be difficulties in deploying efficiently the subsidies.

The current analysis is based on partial equilibrium costs. If the pandemic leads to a reduction in formal employment and more individuals transit to the informal sector, cost estimates could be higher.

## Conclusions

The COVID-19 pandemic is imposing many social challenges. In unequal societies it is imperative to protect vulnerable populations to prevent that the COVID-19 response deepens even further these inequalities. A particularly relevant group is formed by families with children subsisting from the informal economy, as they lack the social security safety-nets available to those in the formal employment sector. Moreover, protecting young children is crucial in the Mexican case given that social protection for this subpopulation is completely absent from current policies. Although scarce resources will be needed for many COVID-19 related responses, investing in the well-being of young children is an imperative allocation due to its long-term consequences and social returns on this investment [7].

## Data Availability

The datasets generated during and/or analyzed during the current study are available in the National Institute of Statistics and Geography (INEGI) repository, [MEX-INEGI.ESD3.03-ENIGH-2018-NS, https://www.inegi.org.mx/programas/enigh/nc/2018/default.html] [6].

## Abbreviation

COVID-19: SARS CoV-2 virus and it’s clinical expression, Coronavirus disease
